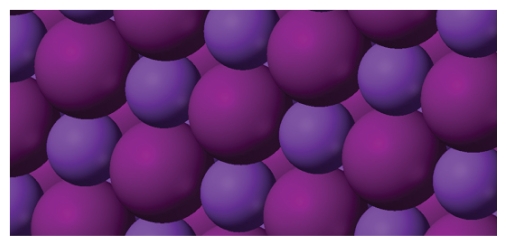# Dietary Iodine: Why are so Many Mothers not Getting Enough?

**DOI:** 10.1289/ehp.118-a438

**Published:** 2010-10

Maternal iodine deficiency has been associated with a number of adverse effects on the infant brain resulting in a continuum of effects depending on the degree of iodine deficiency, from lowered IQ to severe mental retardation.^1^ The thyroid gland uses iodine to make thyroid hormones, which in turn direct brain development.^2^ Insufficient iodine is considered the leading cause of preventable mental retardation in the world, and iodine deficiency in pregnant women has been estimated to result in the loss of some 10–15 IQ points at the global population level.^1^

Data collected over the last 30 years through the National Health and Nutrition Examination Survey (NHANES) suggest iodine levels in the U.S. population, particularly among women of childbearing age, may be getting too low, according to epidemiologist Kevin Sullivan of Emory University. The good news is that, in the past, concerted efforts to ensure adequate iodine intake have yielded beneficial effects. The task now is to understand why so many women are deficient in iodine—and what it will take to make sure pregnant women get enough.

Switzerland, with centuries of serious iodine deficiency, introduced iodized salt in 1922, two years before the U.S. FDA. Unlike in the United States, 60% of Swiss processed foods are made with iodized salt, and almost all the table salt used in Switzerland is iodized. Switzerland monitors iodine levels in the population once every five years and adjusts salt iodine levels accordingly. In 1998 the country increased the salt iodine level in response to reports of marginal iodine status among pregnant women and schoolchildren.^30^ The response was a corresponding rise in urinary iodine to “clearly sufficient” status, and also a more normal level of thyroid function in newborn infants.

## A Downward Turn

Median urinary iodine^3^ among the general U.S. population plummeted by almost 50% between NHANES I (1971–1974) and NHANES III (1988–1994), and the percentage of women with median urinary iodine values below 50 μg/L (indicating moderate or severe deficiency) jumped from 1% to 7%.^4^ Data from NHANES 2003–2004 showed that 37.2% of pregnant women sampled had urinary iodine values below 100 μg/L,^5^ the lower cutoff of the World Health Organization (WHO) recommendation for the general population of 100–199 μg/L.^6^

In a more recent analysis incorporating data from NHANES 2005–2006, Sullivan and colleagues found certain groups of U.S. women were at increased risk for iodine deficiency.^7^ These included nonpregnant, nonlactating women aged 40–44 and various groups of pregnant women (women aged 15–19 years, women aged 30–39 years, women in the non-Hispanic white and other racial/ethnic group, and those who did not consume dairy products, which are one of the chief dietary sources of iodine). The authors concluded that iodine nutrition among U.S. women of reproductive age has stablized since NHANES III (1988–1994) but that the iodine status of pregnant women overall hovers just above the cutoff for iodine sufficiency.

Exposure to goitrogens—agents that inhibit iodine uptake by the thyroid^8^—could exacerbate the impact of iodine deficiency.^9^ Known goitrogens include perchlorate (found in food and drinking water^10^), nitrate (also found in food and drinking water^11^), and thiocyanate (found in cigarette smoke and in cabbage, brussels sprouts, and other cruciferous vegetables^12^). In a 2006 analysis of NHANES data, researchers at the Centers for Disease Control and Prevention found that for women with lower urinary iodine levels, higher levels of urinary perchlorate were associated with decreases in the thyroid hormone thyroxine (T_4_) and increases in thyroid-stimulating hormone (TSH)—a relationship that would be expected if perchlorate were inhibiting iodine uptake enough to interfere with thyroid hormone production.^13^ The strength of this relationship increased when levels of thiocyanate also were taken into account.

When families prepared [more] meals at home, they used iodized salt. As they evolve toward diets filled with foods prepared out of the home, those foods do not use iodized salt, and the iodine levels subside.

— Richard Hanneman, Salt Institute

A similar observation emerged from the cross-sectional Study of Estrogen Activity and Development, which assessed hormone levels of full-term infants over the first 12 months of life.^14^ Infants with urinary iodine under 100 μg/L and higher urine perchlorate levels tended to have higher TSH, although they did not have lower T_4_—possibly because a clear relation between higher T_4_ and lower TSH may not yet be well developed in infants. The authors also found a relationship between urinary iodine and thiocyanate, and thiocyanate also was more strongly related to TSH than perchlorate.

## Why Are We Deficient?

Iodine deficiency is not a new affliction. There is evidence that peoples as far back as the Paleolithic Age suffered from it—some scholars even believe the famous Venus of Willendorf figurine may depict the classic morphology of severe iodine deficiency.^15^ Iodine-rich kelp has been used to treat goiter for thousands of years.^16^ In 1924 the U.S. Food and Drug Administration (FDA) introduced a voluntary program for adding iodine to table salt. The program was hugely successful as evidenced by the elimination of the so-called “goiter belt” throughout the Great Lakes, Appalachian, and Northwestern regions of the United States,^4^ but iodine levels in the United States nevertheless have declined in recent decades (as in many developed countries with long-standing iodization programs), according to Michael Zimmermann, a nutritionist at the Swiss Federal Institute of Technology Zürich.

Experts in this field agree one important change leading to this decline is the increasing trend of eating out and the growing reliance on processed foods. Very often, restaurants and fast food outlets use noniodized salt, according to Richard Hanneman, president emeritus of the Salt Institute, a manufacturers’ group. And virtually no U.S. processed foods are made using iodized salt. “When families prepared [more] meals at home, they used iodized salt,” Hanneman says. “As they evolve toward diets filled with foods prepared out of the home, those foods do not use iodized salt, and the iodine levels subside.”

Most countries that iodize use potassium iodate, which the WHO recommends as a more stable fortificant for salt.^31^ In the United States, other fortificants are used: the FDA limits the fortificant to potassium iodide in the range 0.006–0.01% or copper iodide not to exceed 0.01%.^32^

Salt producers decide whether to iodize salt on the basis of customer specifications, says Hannemann. And there are many misconceptions about the use of iodized salt in food industries, according to Arnold Timmer, a nutrition project officer for the United Nations Children’s Fund (UNICEF). “Some producers think it changes the taste or color of the food, and they do not want to take the risk,” he said in a 2006 UNICEF news article.^17^ “However,” he continued, “many food producers have been using iodized salt for a very long time without any problem.”

A 1995 study corroborated that iodized salt used in typical amounts does not affect the taste or color of foods.^18^ Hanneman says the Salt Institute has just begun an initiative to encourage U.S. food service companies to use iodized salt and to educate food manufacturers about the future need for them to incorporate iodized salt into their products.

In 2007, Boston University School of Medicine associate professor Elizabeth Pearce described other national trends in food production that have changed the amount of iodine we take in.^4^ The amount of iodine in milk declined between the 1970s and 1990s as a result of limits placed on the addition of organic iodine ethylenediamine in cattle feed and disinfectant washes that contain iodine (which is absorbed through the skin and incorporated in cows’ milk). Other changes occurred in commercially baked breads, which once widely used iodate-based bread conditioners to maintain freshness. In 1965 bread contained as much as 150 μg iodine per slice. In 2002, the average iodine content of 17 brands of bread from Boston-area supermarkets was 10 μg per slice, although three varieties of bread contained greater than 300 μg per slice.

## Elusive Iodine

Even when iodized salt is used, the impact can vary widely because of the potential instability of salt’s iodine content. In a 2008 study of salt samples donated by colleagues around the country, chemist Sandy Dasgupta at the University of Texas at Arlington found that 53% of samples from newly opened containers had lower iodine levels than required by FDA labeling regulations.^19^ The study also indicated that salt rapidly lost iodine when the humidity was high, and that samples taken from different depths of the same container could vary in iodine content by 3.3 times, with the most iodine measured toward the bottom of the canister.

Salt intake should be 5 grams a day or less, but all salt consumed should be iodized.

— Michael Zimmermann, Swiss Federal Institute of Technology Zürich

Inconsistent iodine content has shown up in other types of studies. Of the fast food outlets Burger King, McDonalds, Taco Bell and Wendy’s, only Burger King said it used iodized salt when researchers from Boston University Medical Center asked.^20^ But when the researchers compared the iodine content of food items from Burger King and McDonalds to assess the impact of iodized salt in food preparation, the iodine content appeared similar between comparable items from each restaurant. Aside from its fish sandwiches, milkshakes, and the bread in its chicken sandwich, Burger King’s food contained very little measurable iodine.

Prenatal vitamins offer another promising but sometimes disappointing source of iodine. The Institute of Medicine suggests 220 μg iodine intake daily during pregnancy and 290 μg while breastfeeding.^21^ To reach this goal, the American Thyroid Association (ATA) in 2006 recommended that women take prenatal vitamins containing 150 μg iodine daily during pregnancy and lactation to supplement iodine intake from the diet.^22^ But a brief study of the iodine concentrations in 223 prenatal vitamins marketed in the United States found that 49% contained no iodine at all.^23^ Among those that did contain iodine, most claimed to contain the recommended 150 μg or more per daily dose. However, the measured iodine concentration in these vitamins varied by plus or minus 50% from the potency listed on the label.

For vitamins in which the iodine source was potassium iodide, the mean measured iodine content was about 119 μg, or 79% of the labeled value—roughly the percentage of iodine that makes up potassium iodide.^23^ Dasgupta, who in unpublished studies has found similar results using different analytical methods, says this could mean manufacturers erroneously believe that 150 μg potassium iodide is equivalent to 150 μg iodine.

Yet, in the case of table salt, the WHO has stated that under typical circumstances there is about a 20% loss of iodine between salt production and household use.^24^ The WHO recommends that the level at production should account for these losses such that the iodine content in salt at the household be 15–40 ppm. Similarly, the authors of the vitamin study recommend including at least 197 μg of potassium iodide per daily dose to ensure the vitamins contain the promised 150 μg of supplemental daily iodine.^23^

## What about Hypertension?

Discussions about iodization of salt usually spread to include the issue of salt intake and hypertension. Globally, high blood pressure is a major factor in chronic diseases including stroke, coronary heart disease, heart failure, and renal disease.^25^ An estimated 28–30% of the U.S. population has hypertension.^26^ Women with preexisting hypertension face special risks during pregnancy, including the development of preeclampsia and separation of the placenta from the uterine wall.^27^

Americans average a daily intake of more than 3,400 mg of sodium, equivalent to 8.5 g (1.5 teaspoons) salt, most of which comes from processed food.^28^ This substantially exceeds the existing maximum intake level of 2,300 mg sodium, or 5.8 g salt (about 1 teaspoon), established by the 2005 *Dietary Guidelines for Americans*.^28^ In spring 2010 a committee of the Institute of Medicine issued recommendations for ways to reduce U.S. sodium intake, with modifications to standards for sodium content of processed foods listed as a primary strategy.^28^

The goal of reducing salt intake and universal salt iodization are entirely compatible, says Zimmermann. He coordinated a 2007 meeting sponsored by the WHO to discuss the joint goals of reducing hypertension and reducing iodine deficiencies. Participants at the meeting concluded that promoting iodized salt does not conflict with recommending reduced salt intake.^29^ “Salt intake should be five grams a day or less, but all salt consumed should be iodized,” says Zimmermann.

Sullivan and Pearce agree. “There is a need in the United States to reduce overall salt intake. It would seem prudent to recommend that most people reduce their salt intake, and of the salt they do consume, it should be iodized,” Sullivan says.

But, pragmatically, Pearce says that requiring universal salt iodization in this country is very unlikely to happen. “An effort to mandate salt iodization in the United States back in the 1940s was met with significant opposition,” she notes. In 2008 Dasgupta described that event, writing, “Veteran congresswoman Frances Bolton once attempted to legislate mandatory salt iodization, the salt producers association prevailed with the argument that this is medication by legislation. Failing mandatory iodization, the U.S. Public Health Service launched a nationwide educational program in 1949 for consumers to ask specifically for iodized salt at the grocery. All of these have fallen by the wayside.”^19^

In the absence of such a large-scale public health intervention, Pearce thinks the ATA’s approach for prenatal vitamins is the correct way to approach the problem of insufficient iodine in pregnancy. “The problems have been that too few people know of the ATA recommendations, and consumers may have difficulty in finding iodine-containing prenatal multivitamins,” she says. The ATA is working with various obstetric and other societies at present to try to expand knowledge about the current recommendations.

## Figures and Tables

**Figure:**
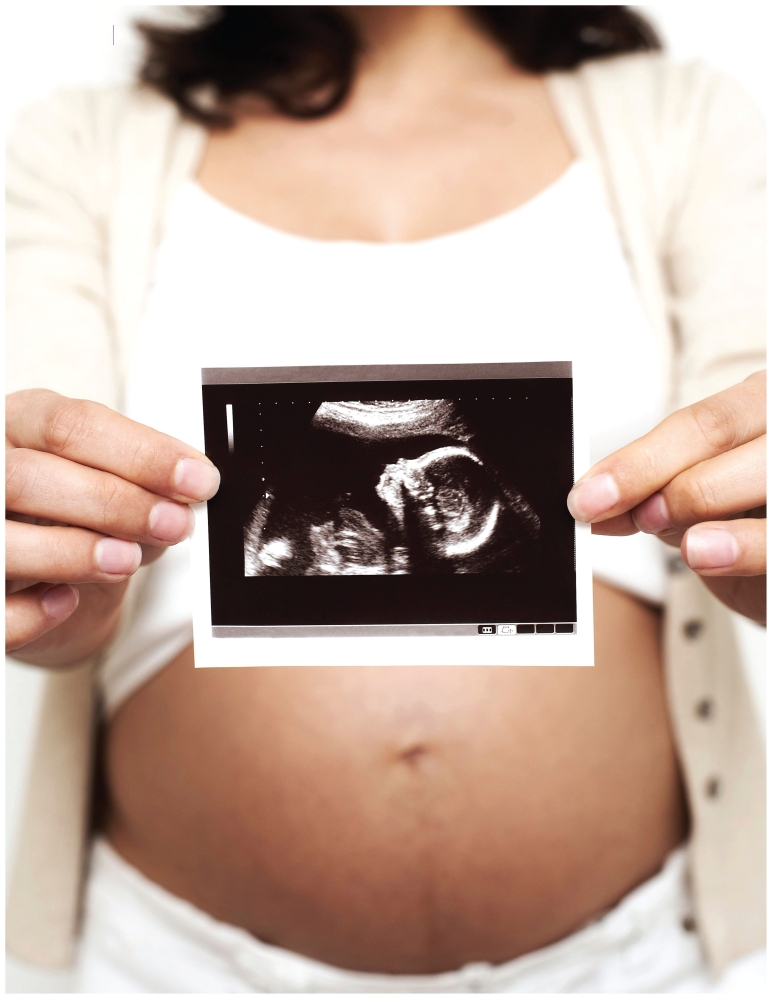
Fortification of foods such as salt has been shown to be an effective way to ensure pregnant women get adequate iodine, a critical nutrient for proper brain growth. But dietary and food production shifts in the past few decades have resulted in dramatically decreased population levels of iodine, with potentially devastating effects for babies of iodine-deficient mothers.

**Figure:**
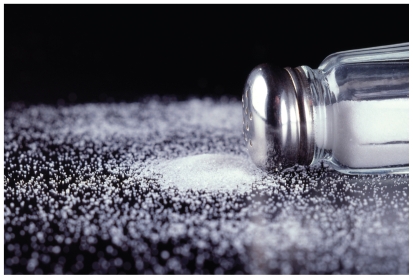


**Figure:**
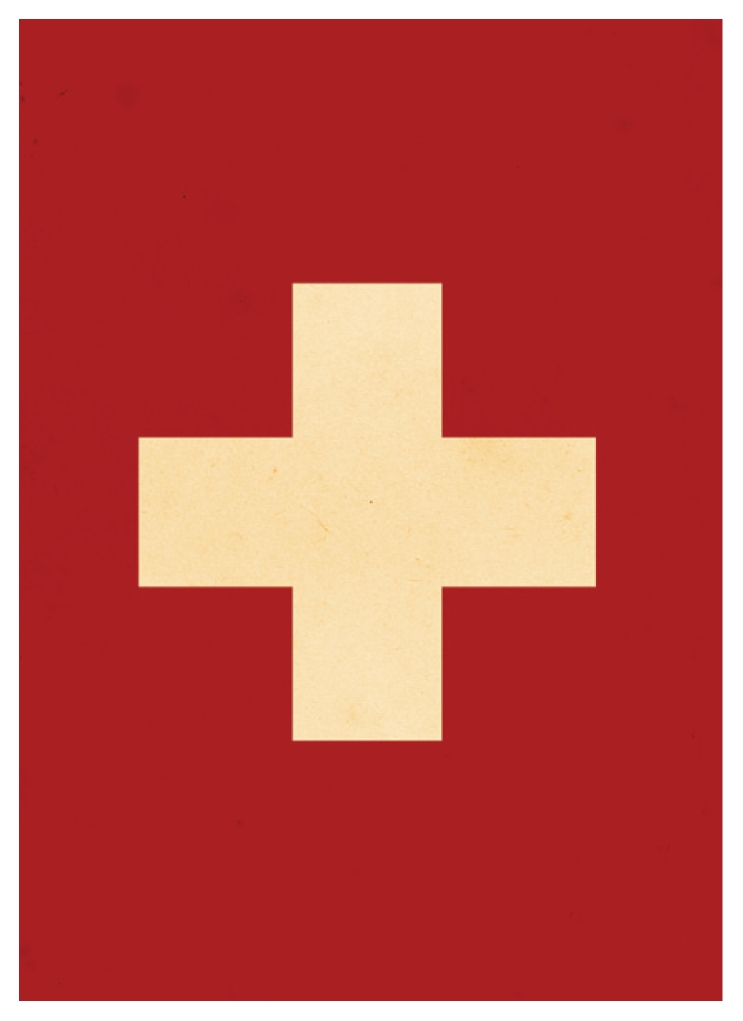


**Figure:**